# Rhodomollins A and B, two Diterpenoids with an Unprecedented Backbone from the Fruits of *Rhododendron molle*

**DOI:** 10.1038/srep36752

**Published:** 2016-11-14

**Authors:** Yong Li, Yun-Bao Liu, Hui-Min Yan, Yang-Lan Liu, Yu-Huan Li, Hai-Ning Lv, Shuang-Gang Ma, Jing Qu, Shi-Shan Yu

**Affiliations:** 1 State Key Laboratory of Bioactive Substance and Function of Natural Medicines, Institute of Materia Medica, Chinese Academy of Medical Sciences and Peking Union, Medical College, Beijing 100050, People’s Republic of China; 2 Institute of Medicinal, Biotechnology, Chinese Academy of Medical Sciences and Peking Union Medical, College, Beijing 100050, People’s Republic of China

## Abstract

Two new grayanoids, rhodomollin A (**1**) and rhodomollin B (**2**), possessing an unprecedented D-*homo* grayanane carbon skeleton, were isolated from the fruits of *Rhododendron molle*. The structures of **1** and **2** were fully characterized using a combination of spectroscopic analyses and X-ray crystallography. Rhodomollin B (**2**) exhibited modest activity against influenza virus A/95-359, with an IC_50_ value of 19.24 *μ*M.

Grayanoids are characteristic metabolites of Rhododendron plants and possess diverse structures and significant biological activities^1^. In particular, grayanoids with a wide spectrum of biological activities, such as sodium-channel-modulating^2^, analgesic^3^, sedative^4^, and insect antifeedant activities^5^, have been isolated from plants of the Rhododendron genus in recent years. Ten types of grayanane-related carbon skeletons have been reported, including grayanane (A-*nor*-B-*homo ent*-kaurane)^6^, 1,5-secograyanane^7^, 3,4-secograyanane^8^, 9,10-secograyanane^9^, 1,10:2,3-disecograyanane^10^, leucothane (A-*homo*-B-*nor* grayanane)^11^, kalmane (B-*homo*-C-*nor* grayanane)^12^, 1,5-seco kalmane^13^, micranthane (C-*homo* grayanane)^14^, and mollane (C-*nor*-D-*homo* grayanane)^15^. We previously reported mollolide A, a grayanoid with a new 1,10:2,3-disecograyanane skeleton^10^, and mollanol A, the first example with a C-*nor*-D-*homo* grayanane carbon skeleton^15^, from *Rhododendron molle* G. Don, a plant that is used in traditional Chinese medicine as an anodyne and anesthetic^16^. In our continuing efforts to identify structurally interesting and biologically important grayanoids, two new grayanoids, rhodomollin A (**1**) and rhodomollin B (**2**), possessing an unprecedented D-*homo* grayanane (featured a 5/7/6/6-fused ring system) carbon skeleton, were isolated from the fruits of *R. molle*. Herein, the isolation, structural elucidation, and evaluation of the anti-viral activity of these compounds are discussed.

## Results and Discussion

**Figure 1:**
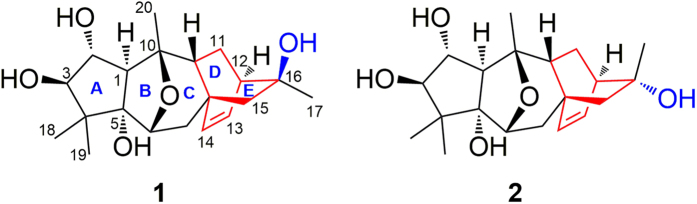
Structures of compounds **1** and **2**.

Rhodomollin A (**1**) was obtained as a colorless crystal, and its molecular formula was determined to be C_20_H_30_O_5_ with six degrees of unsaturation based on HRESIMS analysis (*m/z* 373.1988 [M + Na]^+^, calcd 373.1985), consistent with the ^1^H and ^13^C NMR data. The IR spectrum included a strong absorption band at 3424 cm^−1^, indicative of the presence of hydroxy group(s). Analysis of the ^13^C NMR (DEPT) data led to the assignment of four methyls, three methylenes, eight methines (three oxygenated, two olefinic carbons), and five quaternary carbons (three oxygenated) (see Table 1). The two olefinic carbons accounted for only one of the six degrees of unsaturation; thus, five rings should be present. Detailed analysis of the ^1^H and ^13^C data of **1** indicated that **1** shares partial structural features (rings A, B and C in Fig. 1) with previously reported grayanane-type diterpenoids.

**Figure 2:**
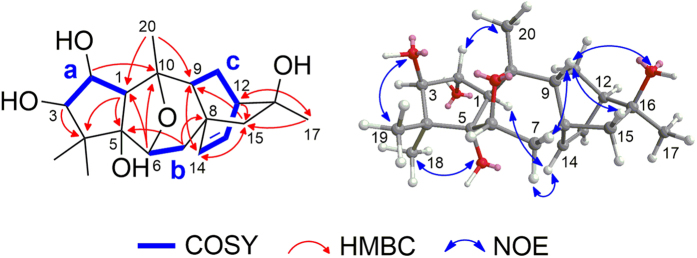
Selected ^1^H-^1^H COSY, HMBC, and NOESY correlations for compound **1**.

The two gem-dimethyl singlets (*δ*_H_ 1.51, H_3_-18; 1.47, H_3_-19) were chosen as the starting point for the structural elucidation of ring A. The HMBC correlations from H_3_-18/H_3_-19 to C-3/C-4/C-5, from H-2 to C-5, and from H-3 to C-1 (see Fig. 2) and the assignments of fragment a [C(1)H−C(2)H−C(3)H] by ^1^H−^1^H COSY and HSQC established the five-membered ring A.

Ring B was elucidated by the HMBC correlations from H-6 to C-1, C-5 and C-10, from H_3_-20 to C-1 and C-10, and from H-2 to C-1, C-5, and C-10. Because the five oxygen atoms in the molecular formula accounted for the six oxygenated carbons in the ^13^C-NMR spectrum and H-6 was correlated to C-10 in the HMBC spectrum, ring B was determined to be a furan ring formed through a C6-O-C10 oxygen bridge and fused with ring A at C-1 and C-5. The HMBC correlations from H_3_-20 to C-9 and from H_2_-7 to C-8/C-9 together with spin system b, C(6)H-C(7)H_2_, indicated that ring C shares the C6-O-C10 oxygen bridge with ring B to form a pyran ring.

The HMBC correlations from H-14 to C-8 and C-9 and from H-9 to C-14, together with spin system c elucidated from the ^1^H-^1^H COSY and HSQC spectra, established a six-membered ring (ring D in Fig. 1). In addition, the HMBC correlations from H_2_-15 to C-8, C-9, and C-14 indicated the linkage of C-15 and C-8. The HMBC correlations from H-12 to C-16 and C-15 confirmed the connectivity of C-16 and C-12. Thus, another six-membered ring (ring E) was established. Subsequently, the HMBC correlations from H_3_-17 to C-12, C-15, and C-16 unambiguously placed CH_3_-17 on C-16.

Consequently, the planar structure of **1** was determined and features a 5/5/6/6/6-fused ring system. Rings D and E compose a bicyclo[2.2.2]octane ring system in which C-8 and C-12 are bridged by C-15 and C-16. This is the first example of a D-*homo* grayanane carbon skeleton.

The relative configurations were determined by NOESY correlations (Fig. 2). NOESY correlations of H-3/H-1 and OH-3/H_3_-19 revealed the *α*-orientation of H-1 and H-3. NOESY correlations between H-1 and H-14 confirmed that the C14-C8 bond was also *α*-oriented, indicating that bond C8-C15 is *β*-configured. Additionally, H-12 must adopt an *α*-orientation in the rigid bicyclo[2.2.2]octane ring system formed by rings D and E. NOESY correlations of H-7b/H-9, H-9/H-15a, and H-9/OH-16 indicated that H-9, OH-16, H-15a, and H-7b are on the same side with a *β*-configuration.

**Figure 3:**
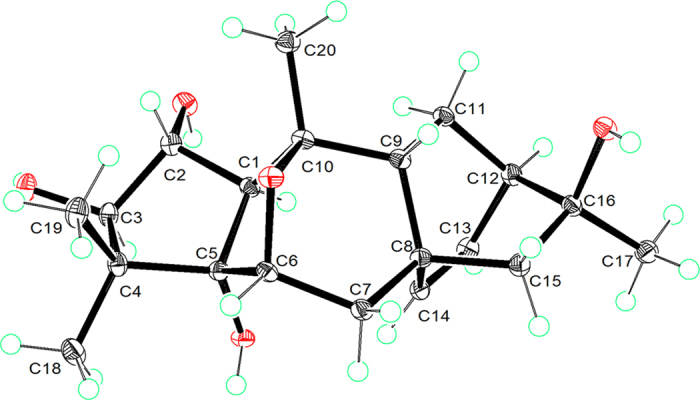
X-ray structure of compound **1**.

The NOESY correlations of OH-5/H-1 and OH-5/H_3_-18 suggested that the two five-membered rings A and B were *cis*-fused to form a bicyclo[3.3.0]octane. The *cis*-fusion of rings A and B is preferred in bicyclo[3.3.0]octane because the *trans*-fusion of two five-membered rings would involve considerable torsional strain, with a very large enthalpy difference (*cis* to *trans* 29.6 kJ/mol)^17^. Due to a lack of NOE evidence, the relative configuration of the C6-O-C10 oxygen bridge could not be assigned directly by NOESY. Fortunately, a high-quality single crystal of **1** was obtained from a methanol-water solvent system. The X-ray crystallographic (Cu Kα radiation) data (Table S1–S4) corroborated the planar structure and the relative configuration of **1** and further allowed the assignment of its absolute configuration as 1*R*, 2*R*, 3*R*, 5*S*, 6*R*, 8*R*, 9*R*, 10*S*, 12*R*, 16*S* [with a Flack parameter of 0.05 (10)] (see Fig. 3). The crystallographic data for **1** have been deposited at the Cambridge Crystallographic Data Centre (CCDC) under deposition number 1445432.

Rhodomollin B (**2**) was obtained as white amorphous powder. Its molecular formula, C_20_H_30_O_5_, as deduced from HRESIMS, is identical to that of **1**. The UV, IR, and NMR spectral data of **2** also resembled those of **1**, except that compared with **1**, the ^13^C NMR signals for C-15 and C-17 in **2** were downshifted 1.5 ppm and upshifted 2.3 ppm, respectively. Careful analysis of the spectroscopic data allowed us to conclude that its planar structure was identical to that of **1**.

The NOESY correlations of H-1/H-3, H-1/H-14, H-9/H-7b, H-9/H-15a, and OH-5/H_3_-18 revealed that the two compounds share the same relative configurations in these positions, except the stereochemistry at C-16. The relative configuration of OH-16 was then confirmed to be *α*-oriented on the basis of the NOESY correlation between H-9 (*β*) and H_3_-17, indicating that **2** is a C-16 diastereomer of **1**. Thus, the structure of **2** was established and named rhodomollin B.

**Figure 4:**
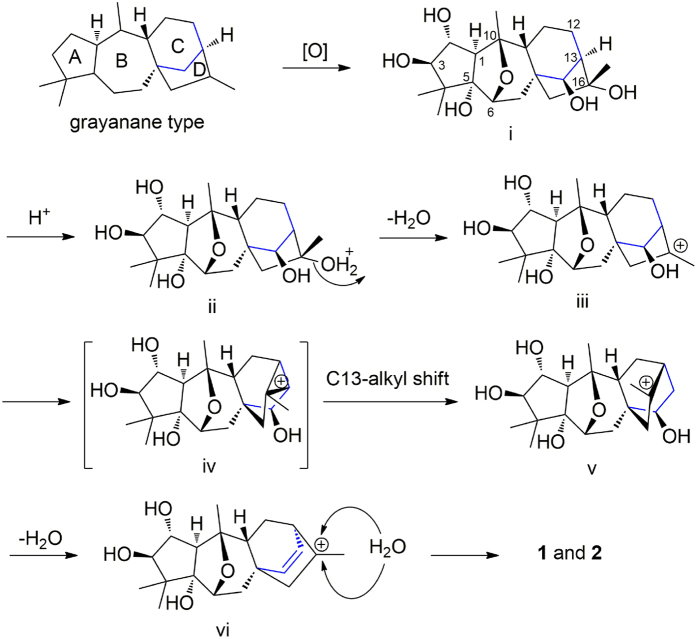
Proposed biosynthetic pathway for 1 and 2.

Rhodomollins A (**1**) and B (**2**) represent a new tetracyclic diterpene carbon skeleton with an unprecedented D-*homo* grayanane (featured a 5/7/6/6-fused ring system) carbon skeleton. We have named this new skeleton “rhodomollane”. Compound **1** possesses an unusual *β-*16-OH. The stereochemistry at C-16 in grayanoids is highly conserved. A likely biosynthetic pathway is proposed in Fig. 4. Compounds **1** and **2** share a common precursor i, which undergoes a C13-alkyl shift to form a carbocation center at C-16^18^. The carbocation center at C-16 can then be attacked by H_2_O from two sites to form diastereomers **1** and **2**.

The anti-viral activities of rhodomollins A (**1**) and B (**2**) were assessed *in vitro* in A/95-359 influenza virus-infected MDCK cells. Rhodomollin B (**2**) exhibited modest activity against influenza virus A/95-359, with an IC_50_ value of 19.24 *μ*M, whereas rhodomollin A (**1**) was inactive (IC_50_ > 100 *μ*M).

## Materials and Methods

### General experimental procedures

Optical rotations were measured with a PE model 343 polarimeter. CD spectra were recorded on a JASCO-815 CD spectrometer. A Nicolet 5700 FT-IR microscope instrument was used to record IR spectra. NMR spectra were obtained on INOVA-500 spectrometer. ESIMS were measured with an Agilent 1100 Series LC/MSD Trap mass spectrometer. HRESIMS data were measured using an Agilent 6520 Accurate-Mass Q-TOF LC/MS spectrometer. X-ray experiments were carried on a Gemini E X-ray single crystal diffractometer. A preparative Shimadazu LC-6AD HPLC equipped with SPD-20A and RID-10A detectors (Kyoto, Japan) along with an YMC ODS-A column (250 × 20 mm, 5 *μ*m, Kyoto, Japan) were used to purify the compounds. Macroporous resin (D101 type, The Chemical Plant of NanKai University, China), MCI gel, Mitsubishi chemical corporation, Sepherdex LH-20, GE chemical corporation, Si gel (160–200, 200–300 mesh, Qingdao Marine Chemical Factory, China) and ODS (50 *μ*m, Merck, Germany) were used for column chromatography (CC). TLC was carried out with precoated Si gel plates (Qingdao Marine Chemical Factory, China). Spots were visualized by spraying with 10% EtOH-sulfuric reagent; see supplementary information S4.

### Plant material

The fruits of *Rhododendron molle* were collected in Guangxi Province of China, in 2012. It was authenticated by Guang-Zhao Li, a professor of Guangxi Institute of Botany. A voucher specimen (ID-s-2445) was deposited in the herbarium of our institute (Institute of Materia Medica, Chinese Academy of Medical Sciences); see supplementary information S4.

### Extraction and isolation

Extracts from the dried fruits of *Rhododendron molle* (100 kg) were obtained (2 h per extraction) with EtOH-H_2_O (95:5, v/v) under conditions of reflux. The extract was suspended in 30 L of H_2_O, and then partitioned with petroleum ether, CH_2_Cl_2_, EtOAc and MeOH (three times with 15 L each). The EtOAc fraction was then further separated on a macroporous resin column and eluted in a gradient of H_2_O:EtOH (70:30, 40:60, 5:95, v/v) in order of increasing concentrations of EtOH. The 30% EtOH fraction was further resolved on a MCI gel column and eluted in a gradient of MeOH:H_2_O (1:9–10:0, v/v) to obtain 15 fractions (EM_1_–EM_15_). Fraction EM_9_ was purified by Sephadex LH-20 column to obtain a terpenoid-containing fraction EM_9_G_1_ (9 g), which was further loaded onto an Si gel column and eluted in a gradient of CH_2_Cl_2_:MeOH (20:1–1:2, v/v) to obtain 10 fractions (EM_9_G_1_L_1_–EM_9_G_1_L_10_). EM_9_G_1_L_6_ (0.68 g) was purified by preparative HPLC and semi-preparative HPLC to yield **1** (6.0 mg). EM_9_G_1_L_7_ (0.55 g) was purified by preparative HPLC and semi-preparative HPLC to yield **2** (2.2 mg); see supplementary information S5.

### Rhodomollin A (1)

Colorless crystals; [α]^20^_D_ + 7.1 (*c* 0.1, MeOH); UV (MeOH) λ_max_ (log *ε*) 204.8 (2.86) nm; IR(KBr) *ν*_max_ 3424, 2960, 2932, 1645, 1463, 1418, 1381, 1139, 1057, 1029, 984, 907, 875, 817, 798, 730, 665, 635 cm^−1^. For ^1^H and ^13^C NMR spectroscopic data, see Table 1; HRESI-MS 373.1986 (calcd for C_20_H_30_NaO_5_, 373.1985); see supplementary information S6–S19.

### Rhodomollin B (2)

White powder; [α]^20^_D_ + 8.5 (*c* 0.14, MeOH); UV (MeOH) λ_max_ (log *ε*) 204.0 (2.9) nm; IR(KBr) ν_max_ 3374, 2931, 2872, 1597, 1443, 1379, 1123, 1081, 1050, 1129, 926, 910, 816, 732, 634 cm^−1^. For ^1^H and ^13^C NMR spectroscopic data, see Table 1; HRESI-MS 373.1986 (calcd for C_20_H_30_NaO_5_, 373.1985); see supplementary information S20–S33.

### Anti-influenza A Assays

Madin-Darby Canine Kidney (MDCK) cells and influenza A (A/Hanfang/359/95, H3N2) were from the Institute of Virology, Chinese Academy of Preventive Medicine. Confluent Madin-Darby Canine Kidney (MDCK) cells were infected with influenza A (A/Hanfang/359/95, H3N2) in 96-well microplates. After 2 h of viral adsorption (37 °C), the monolayers were washed by PBS and incubated with or without test compounds in the maintenance medium (37 °C). Cytopathogenic effect (CPE) caused by viral invasion was evaluated when the viral control group reached a level of 4 and the antiviral activity of test compounds was determined according to the Reed and Muench method^19^,^20^,^21^.

The cytotoxicity of compounds in the presence of MDCK cells were monitored by CPE. MDCK cells (2.5 × 10^4^/well) were plated into a 96-well plate. A total of 24 h later, the monolayer cells were incubated in the presence of various concentrations of test compounds. The cells were cultured for 48 h at 37 °C and 5% CO_2_ in a carbon-dioxide incubator. CPE were counted on the infected MDCK cells. Median toxic concentration (TC_50_) values of test compounds were calculated by the method of Reed and Muench. Compounds **1** and **2** showed no cytotoxicicy in this assay (TC_50_ > 100 *μ*M).

## Additional Information

**How to cite this article**: Li, Y. *et al.* Rhodomollins A and B, two Diterpenoids with an Unprecedented Backbone from the Fruits of *Rhododendron molle. Sci. Rep.* **6**, 36752; doi: 10.1038/srep36752 (2016).

**Publisher’s note:** Springer Nature remains neutral with regard to jurisdictional claims in published maps and institutional affiliations.

## Supplementary Material

Supplementary Information

## Figures and Tables

**Table 1:** NMR data for compounds 1 and 2 in C_5_D_5_N (*J* in Hz).

No.	1		2	
	*δ*_H_	*δ*_C_	*δ*_H_	*δ*_C_
1	2.90 (d, 6.0)	57.0	2.88 (d, 6.0)	56.8
2	4.65 (m)	78.0	4.65 (m)	78.0
3	4.66 (m)	84.8	4.66 (m)	84.8
4	—	47.3	—	47.3
5	—	89.3	—	89.2
6	4.36 (brd, 4.5)	76.9	4.34 (brd, 4.5)	76.9
7	a 2.57 (d, 13.5) b 1.92 (dd, 13.5, 4.5)	37.9	a 2.55 (m) b 1.86 (m)	38.1
8	—	37.6	—	37.8
9	2.75 (m)	49.4	2.07 (m)	49.1
10	—	83.5	—	83.4
11	a 2.76 (m) b 1.13 (m)	28.5	a 2.04 (m) b 1.13 (m)	28.9
12	2.50 (m)	44.2	2.56 (m)	44.4
13	6.17 (dd, 8.0, 7.0)	131.4	4.32 (dd, 8.0, 6.5)	131.6
14	7.17 (d, 8.0)	141.3	7.27 (d, 8.0)	140.2
15	a 1.81 (d, 12.0) b 1.54 (d, 12.0)	57.7	a 1.84 (d, 12.0) b 1.59 (d, 12.0)	59.2
16	—	73.4	—	73.3
17	1.34 (s)	33.0	1.55 (s)	30.7
18	1.51 (s)	23.8	1.51 (s)	23.7
19	1.47 (s)	18.4	1.48 (s)	18.4
20	1.54 (s)	22.1	1.53 (s)	22.0
OH-2	6.52 (d, 4.0)	—	6.53 (brs)	—
OH-3	6.66 (d, 5.0)	—	6.68 (brs)	—
OH-5	6.22 (s)	—	6.23 (s)	—
OH-16	5.66 (s)	—		

Measured at 500 (^1^H) and 125 (^13^C) MHz. Overlapping signals are reported without designating multiplicity.
