# Supplementary material for: Rhodomollins A and B, two Diterpenoids with an Unprecedented Backbone from the Fruits of Rhododendron molle
Source: Sci Rep. 2016 Nov 14;6:36752. doi: 10.1038/srep36752 (PMC5107939; doi:10.1038/srep36752)
Supplement: Supplementary Information [file srep36752-s1.doc]

Rhodomollins A and B, two Diterpenoids with an Unprecedented Backbone from the Fruits of *Rhododendron molle*

Yong Li,^†^ Yun-Bao Liu,^†^ Hui-Min Yan,^†^ Yang-Lan Liu,^†^ Yu-Huan Li,^‡^ Hai-Ning Lv,^†^ Shuang-Gang Ma,^†^ Jing Qu, ^†^ and Shi-Shan Yu^†^

^†^State Key Laboratory of Bioactive Substance and Function of Natural Medicines, Institute of Materia Medica, Chinese Academy of Medical Sciences and Peking Union Medical College, Beijing 100050, People’s Republic of China.

^‡^Institute of Medicinal Biotechnology, Chinese Academy of Medical Sciences and Peking Union Medical College, Beijing 100050, People’s Republic of China

**Supplementary Information**

**List of Contents**

1. **Experimental Procedures----------------------------------------------------------------------------S4**
2. **Spectral information of rhodomollin A (1)**
   1. **The UV spectrum of rhodomollin A (1)------------------------------------------------------S6**
   2. **The IR spectrum of rhodomollin A (1)------------------------------------------------------S7**
   3. **(+)-HRESIMS data of rhodomollin A (1)---------------------------------------------------S8**
   4. **The ^1^H NMR spectrum of rhodomollin A (1) in C_5_D_5_N (500 MHz)-------------------S9**
   5. **The ^13^C NMR spectrum of rhodomollin A (1) in C_5_D_5_N (125 MHz)------------------S10**
   6. **The DEPT spectrum of rhodomollin A (1) in C_5_D_5_N (125 MHz)----------------------S11**
   7. **The ^1^H-^1^H COSY spectrum of rhodomollin A (1) in C_5_D_5_N (500 MHz)-------------S12**
   8. **The HSQC spectrum of rhodomollin A (1) in C_5_D_5_N (^1^H: 500 MHz, ^13^C: 125 MHz)----------------------------------------------------------------------------------------------S13**
   9. **The HMBC spectrum of rhodomollin A (1) in C_5_D_5_N (^1^H: 500 MHz, ^13^C: 125 MHz)----------------------------------------------------------------------------------------------S14**
   10. **The NOESY spectrum of rhodomollin A (1) in C_5_D_5_N (500 MHz)-------------------S18**
3. **Spectral information of rhodomollin B (2)**
   1. **The UV spectrum of rhodomollin B (2)-----------------------------------------------------S20**
   2. **The IR spectrum of rhodomollin B (2)-----------------------------------------------------S21**
   3. **(+)-HRESIMS data of rhodomollin B (2)--------------------------------------------------S22**
   4. **The ^1^H NMR spectrum of rhodomollin B (2) in C_5_D_5_N (500 MHz)------------------S23**
   5. **The ^13^C NMR spectrum of rhodomollin B (2) in C_5_D_5_N (125 MHz)-----------------S24**
   6. **The DEPT spectrum of rhodomollin B (2) in C_5_D_5_N (125 MHz)---------------------S25**
   7. **The ^1^H-^1^H COSY spectrum of rhodomollin B (2) in C_5_D_5_N (500 MHz)------------S26**
   8. **The HSQC spectrum of rhodomollin B (2) in C_5_D_5_N (^1^H: 500 MHz, ^13^C: 125 MHz)---------------------------------------------------------------------------------------------S27**
   9. **The HMBC spectrum of rhodomollin B (2) in C_5_D_5_N (^1^H: 500 MHz, ^13^C: 125 MHz)---------------------------------------------------------------------------------------------S28**
   10. **The NOESY spectrum of rhodomollin B (2) in C_5_D_5_N (500 MHz)-----------------S31**
4. **X-Ray Crystallographic data of rhodomollin A (1)**
   1. **Table S1. Crystal data and structure refinement for rhodomollin A (1)----------S34**
   2. **Table S2.** **Atomic parameters for rhodomollin A (1)-----------------------------------S35**
   3. **Table S3. Atom distances for rhodomollin A (1)----------------------------------------S36**
   4. **Table S4. Bond Angles for rhodomollin A (1)-------------------------------------------S37**

**Experimental Procedures**

**General Experimental Procedures.** Optical rotations were measured on a PE model 343 polarimeter. CD spectra were recorded on a JASCO-815 CD spectrometer. IR spectra were recorded on a Nicolet 5700 FT-IR microscope instrument. 1D- and 2D-NMR spectra were obtained on INOVA-500 spectrometer, in C_5_D_5_N with solvent peaks used as references. ESIMS were measured on an Agilent 1100 Series LC/MSD Trap mass spectrometer. HRESIMS data were measured using an Agilent 6520 Accurate-Mass Q-TOF LC/MS spectrometer. X-ray data were measured on Gemini E X-ray single crystal diffractometer or Rigaku MicroMax 002+ X-ray single crystal diffractometer. Preparative HPLC was performed on a Shimadazu LC-6AD instrument with SPD-20A and RID-10A detectors (Kyoto, Japan) using an YMC Pack ODS-A column (250×20 mm, 5 *μ*m, Kyoto, Japan). Macroporous resin (D101 type, The Chemical Plant of NanKai University, China), MCI gel, Mitsubishi chemical corporation, Sepherdex LH-20, GE chemical corporation, Si gel (160–200, 200–300 mesh, Qingdao Marine Chemical Factory, China) and ODS (50 *μ*m, Merck, Germany) were used for column chromatography (CC). TLC was carried out with glass precoated Si gel GF_254_ plates (Qingdao Marine Chemical Factory, China). Spots were visualized under UV light or by spraying with 10% H_2_SO_4_ in EtOH-H_2_O (95:5, v/v) followed by heating.

**Plant material.** Fruits of *Rhododendron molle* were collected in Guangxi Province, China in 2012 and positively identified by Prof. Guang-Zhao Li of Guangxi Institute of Botany. A voucher specimen (ID-s-2445) was deposited in the herbarium at the Department of Medicinal Plants, Institute of Materia Medica, Chinese Academy of Medical Sciences.

**Extraction and Isolation.** Extracts from the dried fruits of *Rhododendron molle* (100 kg) were obtained (2 h per extraction) with EtOH-H_2_O (95:5, v/v) under conditions of reflux. The extract was suspended in 30 L of H_2_O, and then partitioned with petroleum ether, CH_2_Cl_2_, EtOAc and MeOH (three times with 15 L each). The EtOAc fraction was then further separated on a macroporous resin column and eluted in a gradient of H_2_O:EtOH (70:30, 40:60, 5:95, v/v) in order of increasing concentrations of EtOH. The 30% EtOH fraction was further resolved on a MCI gel column and eluted in a gradient of MeOH:H_2_O (1:9–10:0, v/v) to obtain 15 fractions (EM_1_–EM_15_). Fraction EM_9_ was purified by Sephadex LH-20 column to obtain a terpenoid-containing fraction EM_9_G_1_ (9 g), which was further loaded onto an Si gel column and eluted in a gradient of CH_2_Cl_2_:MeOH (20:1–1:2, v/v) to obtain 10 fractions (EM_9_G_1_L_1_–EM_9_G_1_L_10_). EM_9_G_1_L_6_ (0.68 g) was purified by preparative HPLC and semi-preparative HPLC to yield **1** (6.0 mg). EM_9_G_1_L_7_ (0.55 g) was purified by preparative HPLC and semi-preparative HPLC to yield **2** (2.2 mg).


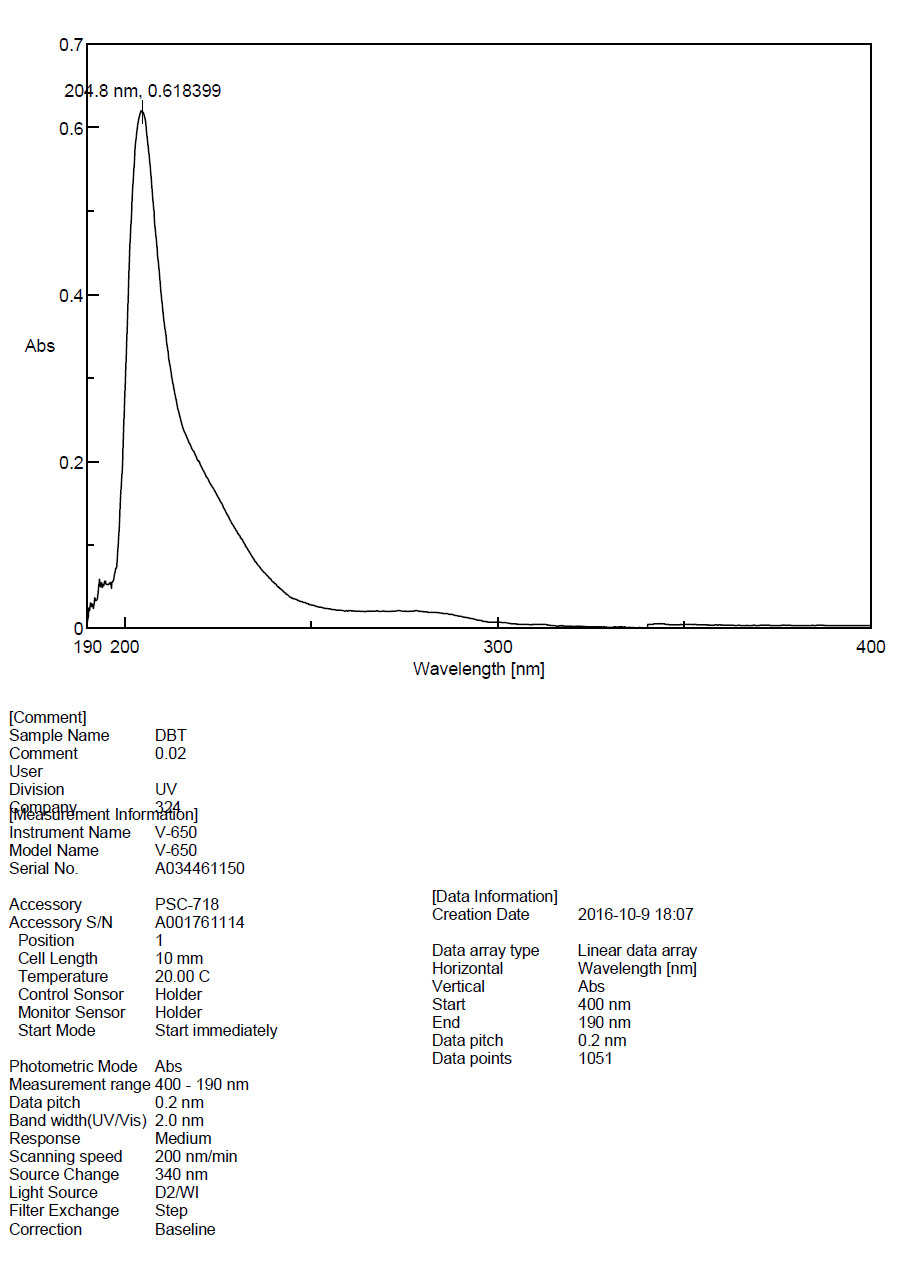


The UV spectrum of rhodomollin A (**1**)


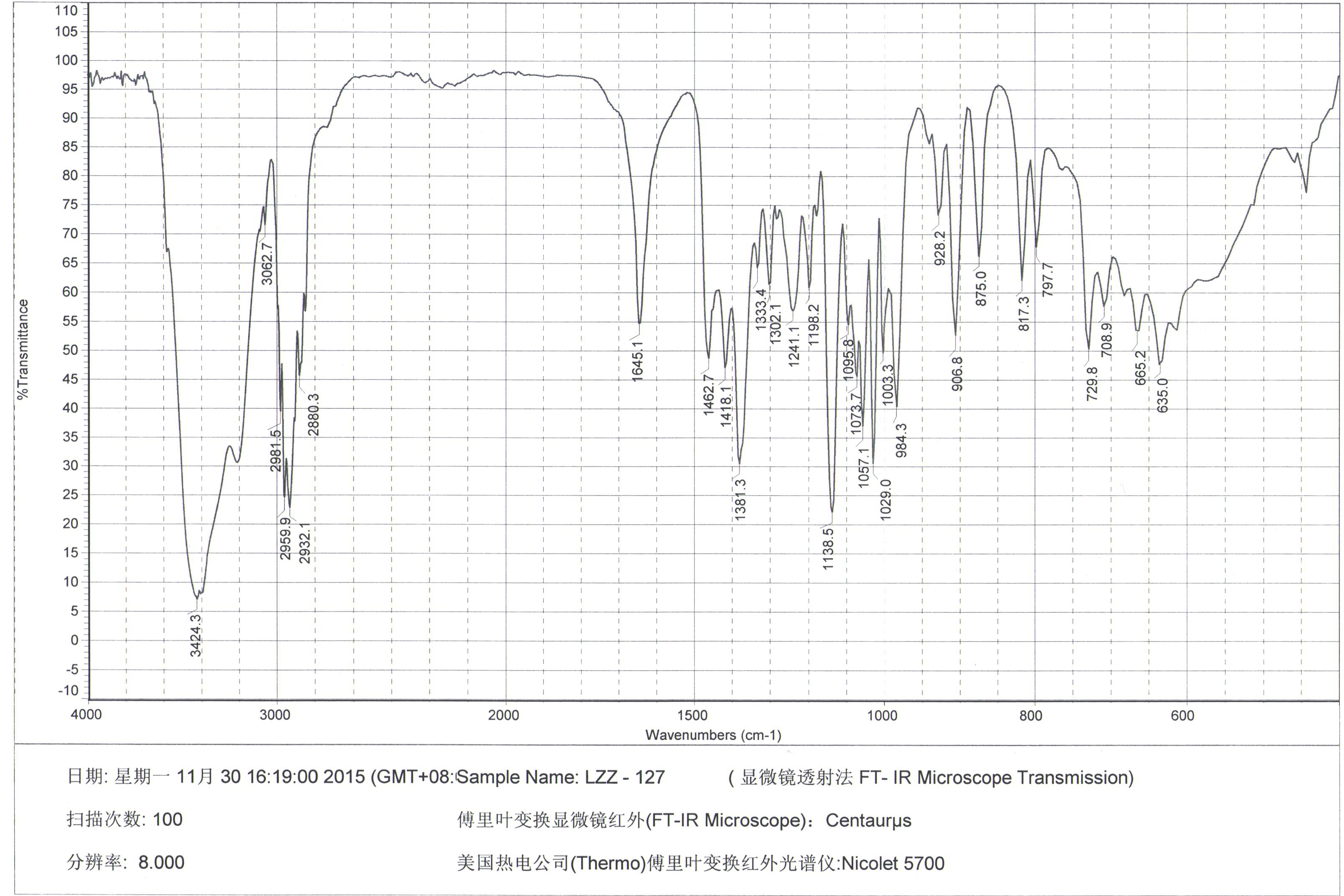


The IR spectrum of rhodomollin A (**1**)


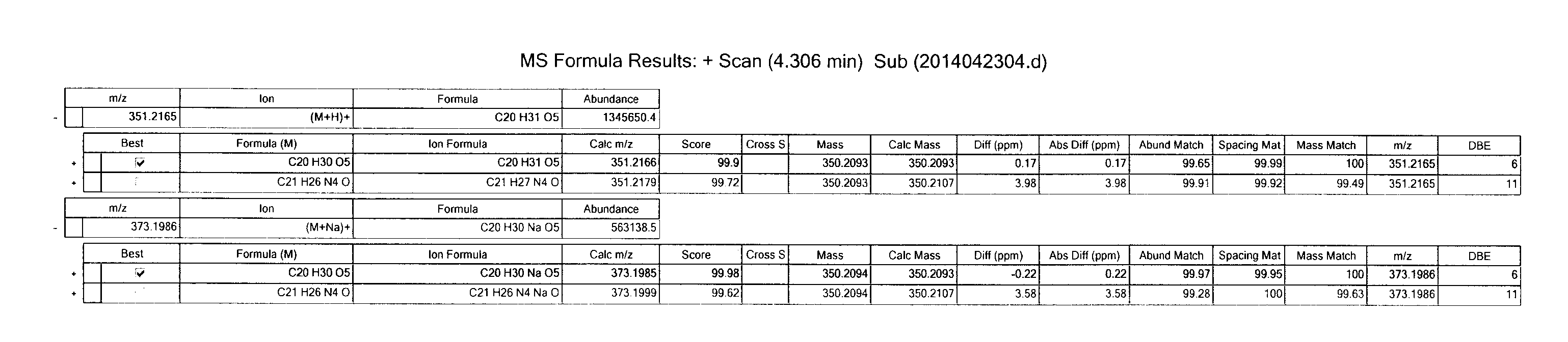


(+)-HRESIMS data of rhodomollin A (**1**)

The ^1^H NMR spectrum of rhodomollin A (**1**) in C_5_D_5_N (500 MHz)

The ^13^C NMR spectrum of rhodomollin A (**1**) in C_5_D_5_N (125 MHz)

The DEPT spectrum of rhodomollin A (**1**) in C_5_D_5_N (125 MHz)

The ^1^H-^1^H COSY spectrum of rhodomollin A (**1**) in C_5_D_5_N (500 MHz)

The HSQC spectrum of rhodomollin A (**1**) in C_5_D_5_N (^1^H: 500 MHz, ^13^C: 125 MHz)

The HMBC spectrum of rhodomollin A (**1**) in C_5_D_5_N (^1^H: 500 MHz, ^13^C: 125 MHz)

The HMBC spectrum (amplified) of rhodomollin A (**1**) in C_5_D_5_N (^1^H: 500 MHz, ^13^C: 125 MHz)

The HMBC spectrum (amplified) of rhodomollin A (**1**) in C_5_D_5_N (^1^H: 500 MHz, ^13^C: 125 MHz)

The HMBC spectrum (amplified) of rhodomollin A (**1**) in C_5_D_5_N (^1^H: 500 MHz, ^13^C: 125 MHz)

The NOESY spectrum of rhodomollin A (**1**) in C_5_D_5_N (500 MHz)


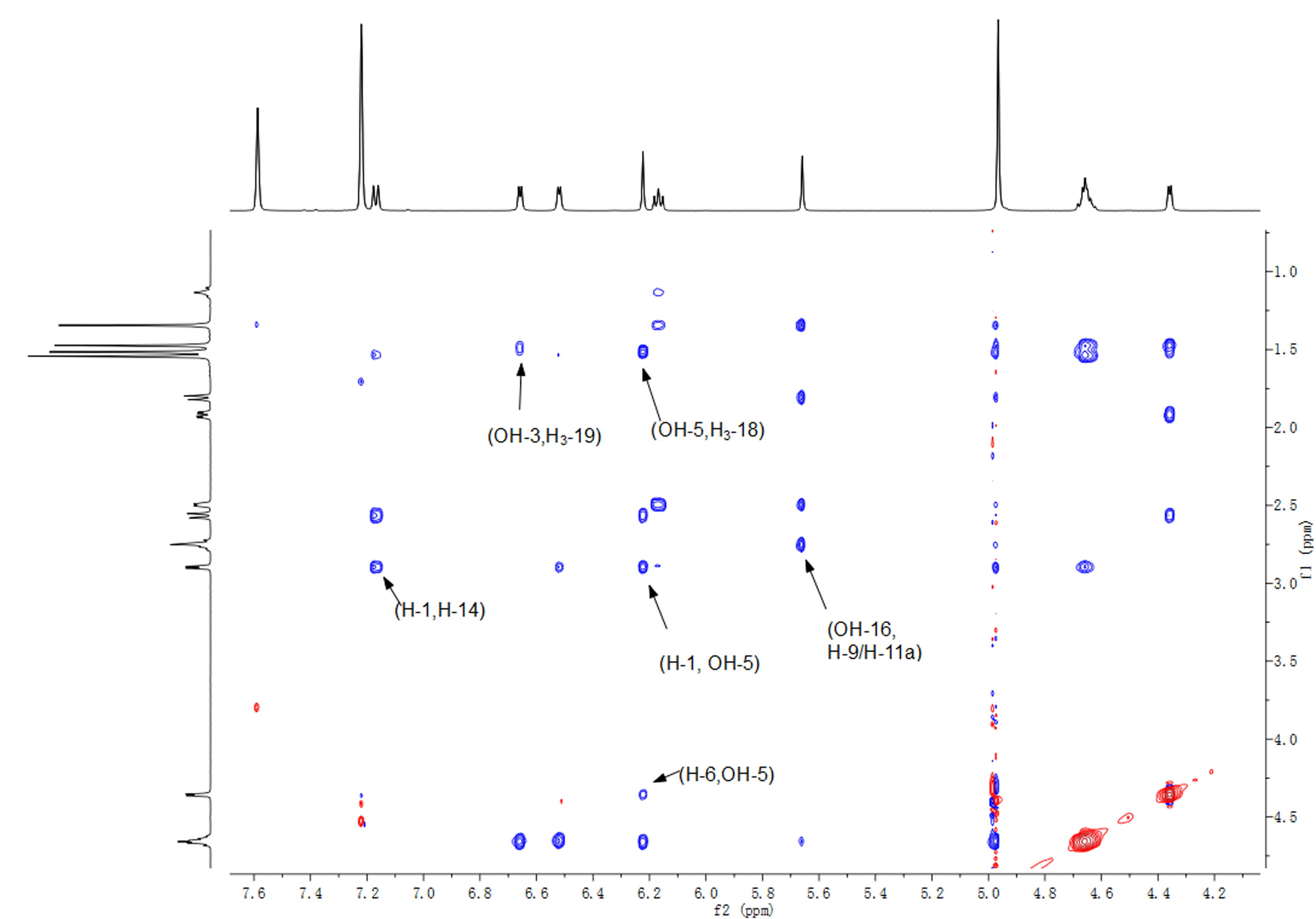


The NOESY spectrum (amplified) of rhodomollin A (**1**) in C_5_D_5_N (500 MHz)


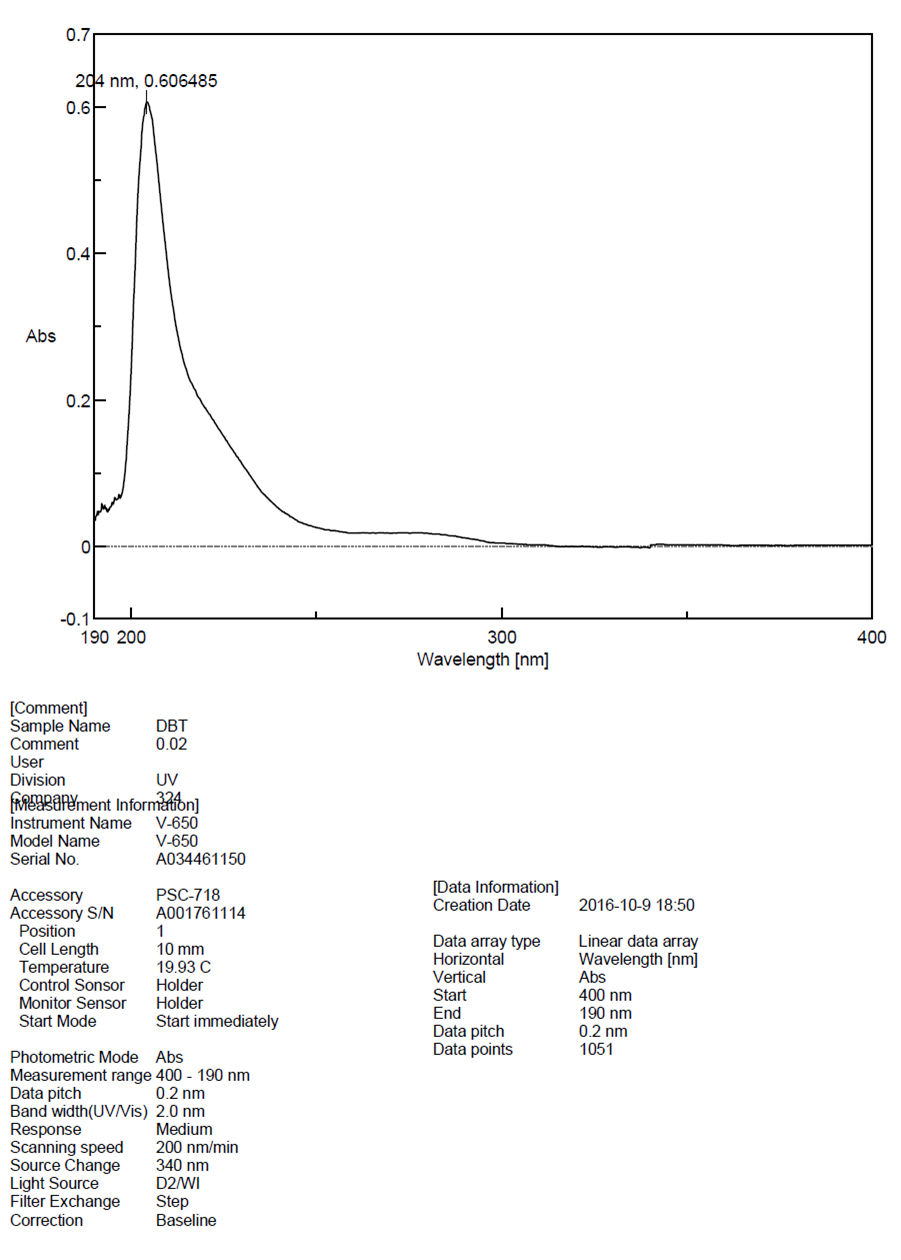


The UV spectrum of rhodomollin B (**2**)


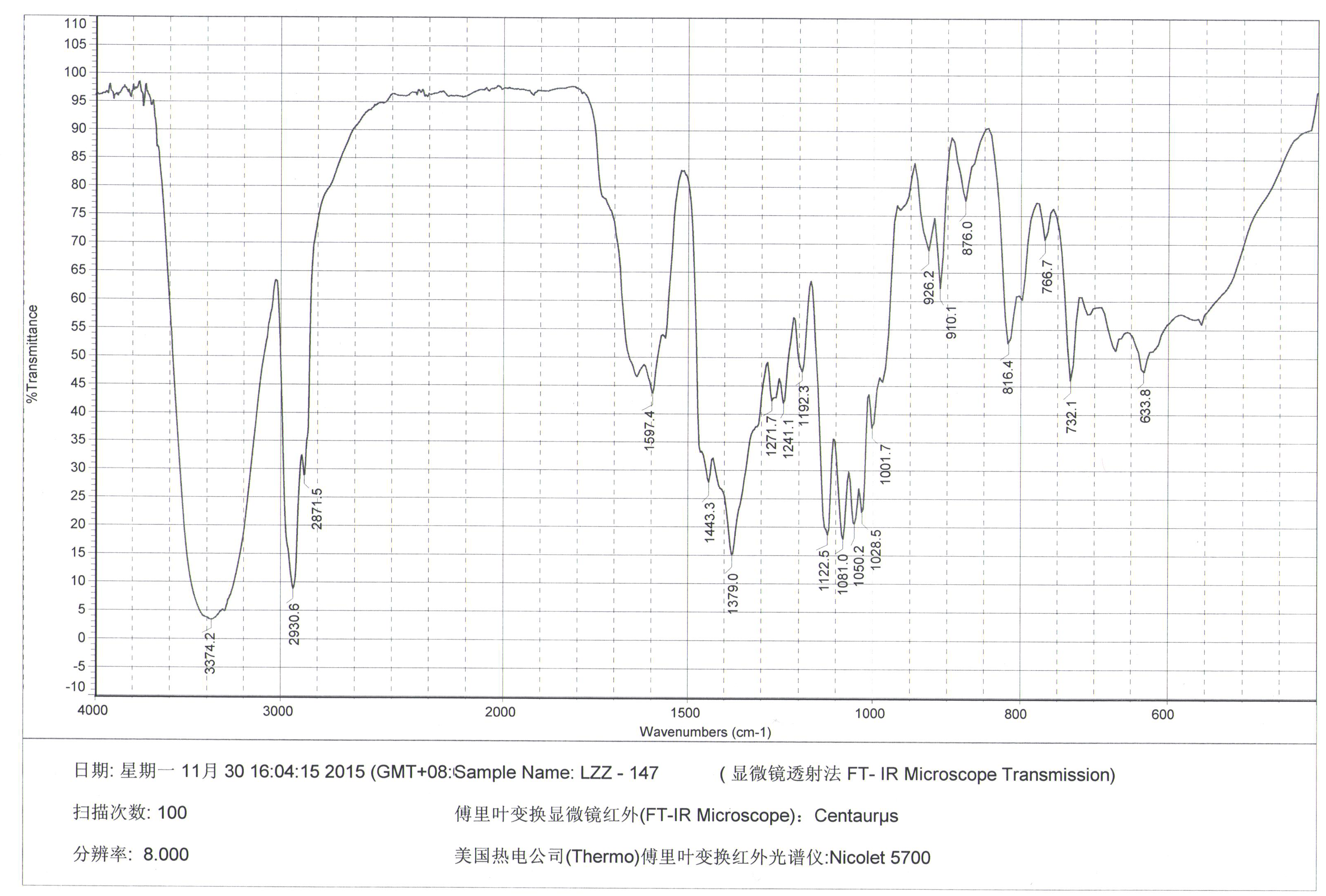


The IR spectrum of rhodomollin B (**2**)


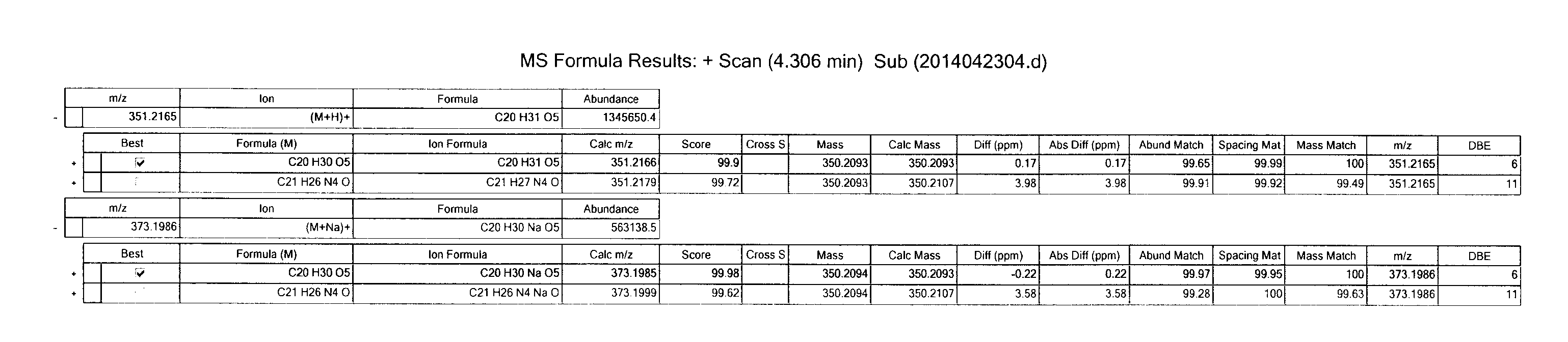


(+)-HRESIMS data of rhodomollin B (**2**)

The ^1^H NMR spectrum of rhodomollin B (**2**) in C_5_D_5_N (500 MHz)

The ^13^C NMR spectrum of rhodomollin B (**2**) in C_5_D_5_N (125 MHz)

The DEPT spectrum of rhodomollin B (**2**) in C_5_D_5_N (125 MHz)

The ^1^H-^1^H COSY spectrum of rhodomollin B (**2**) in C_5_D_5_N (500 MHz)

The HSQC spectrum of rhodomollin B (**2**) in C_5_D_5_N (^1^H: 500 MHz, ^13^C: 125 MHz)

The HMBC spectrum of rhodomollin B (**2**) in C_5_D_5_N (^1^H: 500 MHz, ^13^C: 125 MHz)

The HMBC spectrum (amplified) of rhodomollin B (**2**) in C_5_D_5_N (^1^H: 500 MHz, ^13^C: 125 MHz)

The HMBC spectrum (amplified) of rhodomollin B (**2**) in C_5_D_5_N (^1^H: 500 MHz, ^13^C: 125 MHz)

The NOESY spectrum of rhodomollin B (**2**) in C_5_D_5_N (500 MHz)


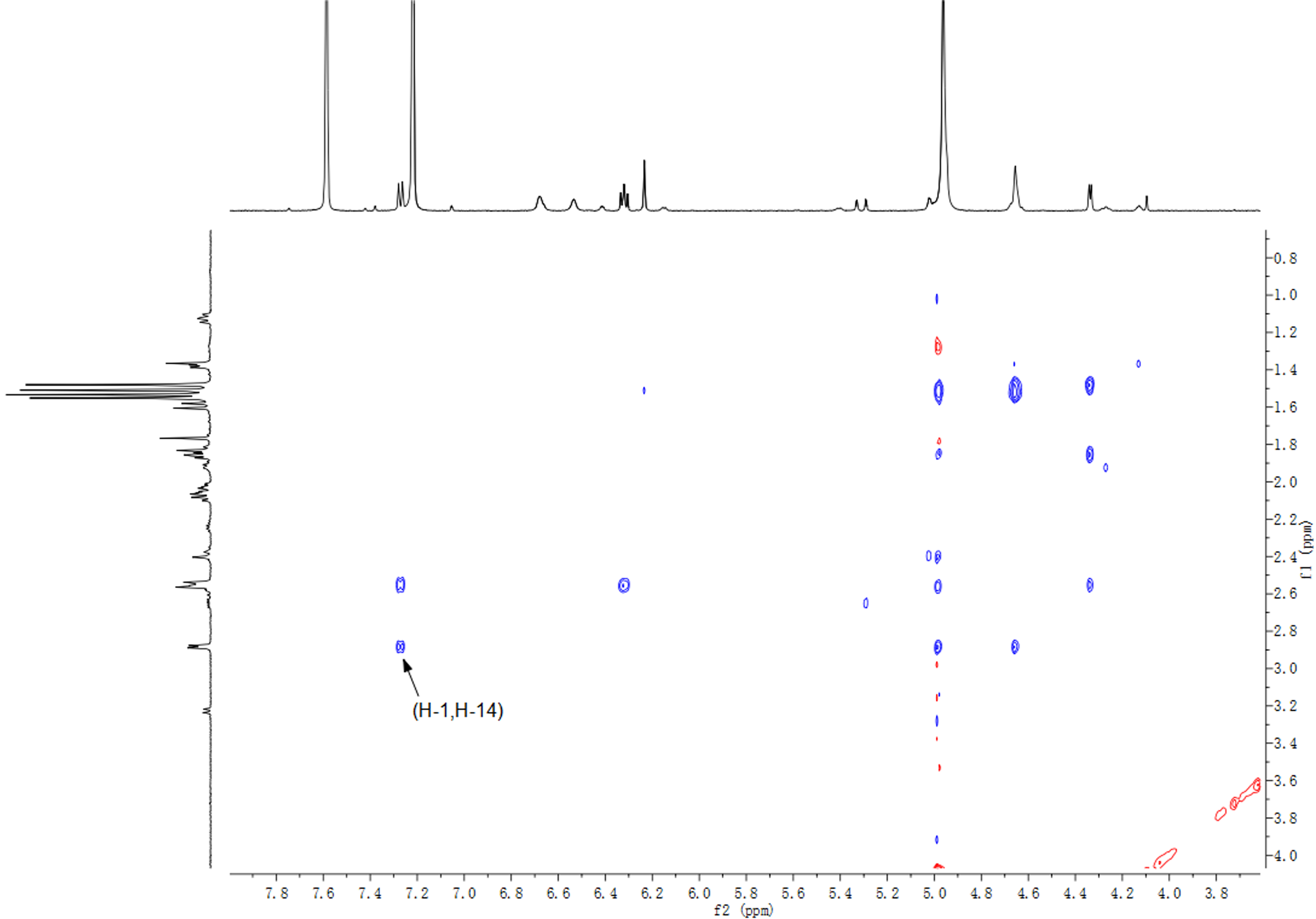


The NOESY spectrum (amplified) of rhodomollin B (**2**) in C_5_D_5_N (500 MHz)

**
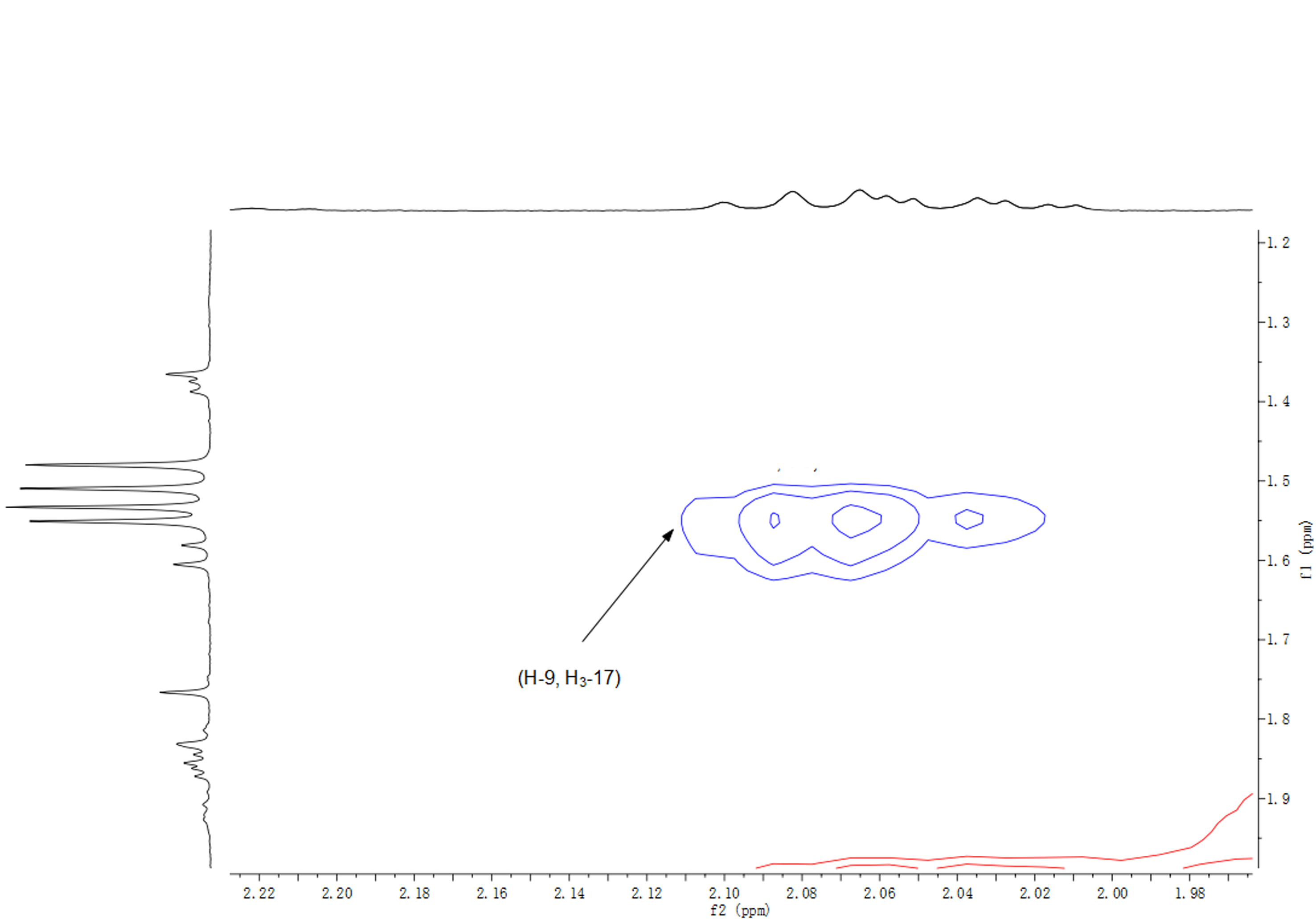
**

The NOESY spectrum (amplified) of rhodomollin B (**2**) in C_5_D_5_N (500 MHz)

**Table S1.** Crystal data and structure refinement for **1**.

| Identification code | 1 |
| --- | --- |
| Empirical formula | C_20_H_30_O_5_ |
| Formula weight | 368.45 |
| Temperature / K | 102.8 |
| Crystal system | monoclinic |
| Space group | P2_1_ |
| a / Å, b / Å, c / Å | 10.2784(2), 8.6949(2), 10.5005(3) |
| α/°, β/°, γ/° | 90, 100.429(2), 90 |
| Volume / Å^3^ | 922.92(4) |
| Z | 2 |
| ρ_calc_ / mg mm^-3^ | 1.326 |
| μ / mm^‑1^ | 0.789 |
| F(000) | 400 |
| Crystal size / mm^3^ | 0.450 × 0.400 × 0.300 |
| 2Θ range for data collection | 8.562 to 142.178° |
| Index ranges | -11≤h≤12, -10≤k≤10, -12≤l≤12 |
| Reflections collected | 6334 |
| Independent reflections | 3415[R(int) = 0.0177 (inf-0.9Å)] |
| Data/restraints/parameters | 3415/1/243 |
| Goodness-of-fit on F^2^ | 1.035 |
| Final R indexes [I>2σ (I) i.e. F_o_>4σ (F_o_)] | R_1_ = 0.0343, wR_2_ = 0.0913 |
| Final R indexes [all data] | R_1_ = 0.0346, wR_2_ = 0.0917 |
| Largest diff. peak/hole / e Å^-3^ | 0.295/-0.217 |
| Flack Parameters | 0.05(10) |
| Completeness | 0.999 |

Crystallographic data of **1** have been deposited at The Cambridge Crystallographic Data Centre and allocated the deposition number CCDC 1445432. The data can be obtained free of charge via www.ccdc.cam.ac.uk/products/csd/ request.

| **Table S2.** Atomic parameters for **1**. | | | | |
| --- | --- | --- | --- | --- |
| Atom | x | y | z | U(eq) |
| O2 | 8502.8(15) | 2787.3(17) | 4477.1(14) | 11.9(3) |
| O1 | 5775.3(14) | 1362.3(17) | 2181.6(15) | 11.8(3) |
| O5 | 6709.1(15) | 4705.4(18) | -1738.4(15) | 12.5(3) |
| O6 | 7881.1(16) | 5028(2) | 6098.6(15) | 17.7(4) |
| O3 | 8728.6(16) | -2061.6(19) | 5321.6(16) | 15.3(3) |
| O4 | 9588.2(16) | -1320.3(19) | 2745.9(15) | 15.4(4) |
| C15 | 9438(2) | 4454(3) | 1113(2) | 13.6(4) |
| C14 | 6815(2) | 5376(3) | 571(2) | 11.9(4) |
| C19 | 6707(2) | -305(3) | 714(2) | 13.6(4) |
| C16 | 8710(2) | 4398(2) | 2033(2) | 11.9(4) |
| C17 | 7583(2) | 934(3) | 6286(2) | 17.3(5) |
| C7 | 7048(2) | 2550(3) | 757.4(19) | 10.2(4) |
| C2 | 7433(2) | 358(3) | 4890(2) | 11.9(4) |
| C6 | 6939.3(19) | 1107(3) | 1573(2) | 10.3(4) |
| C13 | 7582(2) | 5261(3) | -576(2) | 11.3(4) |
| C9 | 6390(2) | 4040(3) | 2632(2) | 11.2(4) |
| C18 | 6143(2) | -579(3) | 4576(2) | 15.7(5) |
| C8 | 7247(2) | 4063(3) | 1565.7(19) | 10.8(4) |
| C3 | 8629(2) | -614(3) | 4677(2) | 12.1(4) |
| C10 | 6238(2) | 2437(3) | 3214.5(19) | 10.6(4) |
| C4 | 8467(2) | -739(3) | 3205(2) | 12.5(4) |
| C20 | 8145(2) | 6805(3) | -888(2) | 14.5(4) |
| C11 | 8094(2) | 2442(3) | -125.2(19) | 11.6(4) |
| C5 | 8072(2) | 914(3) | 2753.8(19) | 10.5(4) |
| C1 | 7548(2) | 1690(3) | 3894.1(19) | 10.4(4) |
| C12 | 8700(2) | 4053(3) | -227(2) | 11.9(4) |

**Table S3. Atom distances for 1.**

| Atom | Atom | Length/Å | Atom | Atom | Length/Å |
| --- | --- | --- | --- | --- | --- |
| O2 | C1 | 1.425(3) | C7 | C11 | 1.544(3) |
| O1 | C6 | 1.471(2) | C2 | C18 | 1.540(3) |
| O1 | C10 | 1.445(3) | C2 | C3 | 1.541(3) |
| O5 | C13 | 1.460(3) | C2 | C1 | 1.579(3) |
| O3 | C3 | 1.424(3) | C6 | C5 | 1.548(3) |
| O4 | C4 | 1.421(3) | C13 | C20 | 1.521(3) |
| C15 | C16 | 1.326(3) | C13 | C12 | 1.551(3) |
| C15 | C12 | 1.513(3) | C9 | C8 | 1.544(3) |
| C14 | C13 | 1.558(3) | C9 | C10 | 1.541(3) |
| C14 | C8 | 1.557(3) | C3 | C4 | 1.527(3) |
| C19 | C6 | 1.517(3) | C10 | C1 | 1.548(3) |
| C16 | C8 | 1.524(3) | C4 | C5 | 1.544(3) |
| C17 | C2 | 1.530(3) | C11 | C12 | 1.545(3) |
| C7 | C6 | 1.535(3) | C5 | C1 | 1.553(3) |
| C7 | C8 | 1.559(3) |  |  |  |

**Table S4.** Bond Angles for **1**.

| Atom 1 | Atom 2 | Atom 3 | Angle 2,1,3/**˚** | Atom 1 | Atom 2 | Atom 3 | Angle 2,1,3/**˚** |
| --- | --- | --- | --- | --- | --- | --- | --- |
| C10 | O1 | C6 | 103.91(15) | C16 | C8 | C14 | 102.79(17) |
| C16 | C15 | C12 | 114.52(19) | C16 | C8 | C7 | 111.17(17) |
| C8 | C14 | C13 | 110.41(17) | C16 | C8 | C9 | 115.35(17) |
| C15 | C16 | C8 | 115.25(19) | C9 | C8 | C14 | 111.42(16) |
| C6 | C7 | C8 | 113.67(17) | C9 | C8 | C7 | 110.34(17) |
| C6 | C7 | C11 | 114.44(18) | O3 | C3 | C2 | 113.97(17) |
| C11 | C7 | C8 | 109.96(17) | O3 | C3 | C4 | 113.82(18) |
| C17 | C2 | C18 | 108.19(18) | C4 | C3 | C2 | 104.01(17) |
| C17 | C2 | C3 | 111.94(18) | O1 | C10 | C9 | 109.25(16) |
| C17 | C2 | C1 | 112.81(19) | O1 | C10 | C1 | 102.44(17) |
| C18 | C2 | C3 | 110.92(18) | C9 | C10 | C1 | 114.83(17) |
| C18 | C2 | C1 | 114.27(17) | O4 | C4 | C3 | 114.97(17) |
| C3 | C2 | C1 | 98.54(16) | O4 | C4 | C5 | 114.30(18) |
| O1 | C6 | C19 | 109.10(16) | C3 | C4 | C5 | 102.66(18) |
| O1 | C6 | C7 | 105.41(17) | C7 | C11 | C12 | 108.60(18) |
| O1 | C6 | C5 | 102.79(15) | C6 | C5 | C1 | 104.76(16) |
| C19 | C6 | C7 | 110.43(17) | C4 | C5 | C6 | 117.71(18) |
| C19 | C6 | C5 | 113.78(18) | C4 | C5 | C1 | 106.04(17) |
| C7 | C6 | C5 | 114.57(17) | O2 | C1 | C2 | 109.57(16) |
| O5 | C13 | C14 | 110.32(17) | O2 | C1 | C10 | 113.02(18) |
| O5 | C13 | C20 | 108.21(17) | O2 | C1 | C5 | 108.33(16) |
| O5 | C13 | C12 | 106.88(17) | C10 | C1 | C2 | 116.71(17) |
| C20 | C13 | C14 | 112.04(18) | C10 | C1 | C5 | 102.76(16) |
| C20 | C13 | C12 | 110.75(17) | C5 | C1 | C2 | 105.65(17) |
| C12 | C13 | C14 | 108.52(17) | C15 | C12 | C13 | 106.82(17) |
| C10 | C9 | C8 | 114.33(17) | C15 | C12 | C11 | 106.57(17) |
| C14 | C8 | C7 | 105.08(16) | C11 | C12 | C13 | 109.89(17) |
